# Mutagenic Potential of*Bos taurus* Papillomavirus Type 1 E6 Recombinant Protein: First Description

**DOI:** 10.1155/2015/806361

**Published:** 2015-12-09

**Authors:** Rodrigo Pinheiro Araldi, Jacqueline Mazzuchelli-de-Souza, Diego Grando Modolo, Edislane Barreiros de Souza, Thatiana Corrêa de Melo, Diva Denelle Spadacci-Morena, Roberta Fiusa Magnelli, Márcio Augusto Caldas Rocha de Carvalho, Paulo Luis de Sá Júnior, Rodrigo Franco de Carvalho, Willy Beçak, Rita de Cassia Stocco

**Affiliations:** ^1^Laboratório de Genética, Instituto Butantan, 05503-900 São Paulo, SP, Brazil; ^2^Programa de Pós-Graduação Interunidades em Biotecnologia, Instituto de Ciências Biomédicas (ICB), Universidade de São Paulo (USP), 05508-900 São Paulo, SP, Brazil; ^3^Laboratório de Biologia Molecular, Genética e Mutagênese, Departamento de Biologia, Faculdade de Ciências e Letras de Assis (FCLA), Universidade Estadual Paulista “Júlio de Mesquista Filho” (UNESP), 19806-900 Assis, SP, Brazil; ^4^Laboratório de Fisiopatologia, Instituto Butantan, 05503-900 São Paulo, SP, Brazil

## Abstract

Bovine papillomavirus (BPV) is considered a useful model to study HPV oncogenic process. BPV interacts with the host chromatin, resulting in DNA damage, which is attributed to E5, E6, and E7 viral oncoproteins activity. However, the oncogenic mechanisms of BPV E6 oncoprotein* per se *remain unknown. This study aimed to evaluate the mutagenic potential of* Bos taurus* papillomavirus type 1 (BPV-1) E6 recombinant oncoprotein by the cytokinesis-block micronucleus assay (CBMNA) and comet assay (CA). Peripheral blood samples of five calves were collected. Samples were subjected to molecular diagnosis, which did not reveal presence of BPV sequences. Samples were treated with 1 *μ*g/mL of BPV-1 E6 oncoprotein and 50 *μ*g/mL of cyclophosphamide (positive control). Negative controls were not submitted to any treatment. The samples were submitted to the CBMNA and CA. The results showed that BPV E6 oncoprotein induces clastogenesis* per se*, which is indicative of genomic instability. These results allowed better understanding the mechanism of cancer promotion associated with the BPV E6 oncoprotein and revealed that this oncoprotein can induce carcinogenesis* per se*. E6 recombinant oncoprotein has been suggested as a possible vaccine candidate. Results pointed out that BPV E6 recombinant oncoprotein modifications are required to use it as vaccine.

## 1. Introduction

Papillomaviruses (PVs) are a group of viruses with epithelium and mucous tropism [1, 2]. PVs can infect all vertebrates, including rabbits [3–5], dogs [6, 7], goats [8], humans [9–12], and bovines [1, 13–15]. In the last decades, an increasing interest in studies involving these viruses has been observed [16, 17]. This fact is justified because the PVs are associated with benign (papillomas) and malignant lesions, which can affect both human [16, 18] and animals [1, 15, 19]. In this scenario,* Bos taurus *papillomavirus is considered the best model for oncogenic process studies associated with PVs [20–23].

*Bos taurus* papillomaviruses, also known as bovine papillomaviruses (BPVs), have a worldwide distribution [23]. It is estimated that 60% of the Brazilian herd is infected by BPV [1]. However, this number can be greater, once the infection can be asymptomatic [24]. BPVs cause bovine papillomatosis, an infectious disease characterized by the presence of multiple papillomas, which can regress spontaneously or progress to malignancy [1]. Among the 14 BPVs types already described [25], BPV-1, BPV-2, and BPV-4 have the most oncogenic potential [21]. BPV-1 and BPV-2 are associated with urinary bladder cancer [26, 27], whereas BPV-4 induces upper gastrointestinal malignancies [28].

Several studies already pointed out that both HPV [29, 30] and BPV [1, 14, 15, 24] interact with the host chromatin, resulting in DNA and chromosomes damage. This damage is induced by the E5, E6, and E7 viral oncoproteins [24]. Although the role of these oncoproteins in the oncogenic process is known [31, 32], there are no studies showing the oncogenic mechanisms of E6 oncoprotein of BPV* per se* to date. The E6 of BPV-1 has 137 amino acids, with four well conserved Cys-X-X-Cys motifs among all PVs [33–35]. Moreover, the E6 oncoprotein has six cysteine residues, which turn the oncoprotein susceptible to oxidation [36]. These characteristics make the production, purification, and obtaining of E6 correct folding difficult [36, 37]. Due to these difficulties, in the last 30 years, different constructions, employing expression vectors in* Escherichia coli*, have been developed in order to produce large quantities of E6 oncoprotein [36–43]. Most of these studies are directed to HPV-16 and BPV-1. In this scenario, Mazzuchelli-de-Souza et al. [44] successfully purified the BPV-1 E6 recombinant protein. A summary of studies involving the E6 oncoprotein is shown in Table 1.

Due the low number of studies about the E6 of BPV to date, the knowledge of oncogenic mechanisms associated with this oncoprotein comes from previous works with E6 of HPV [29, 30]. The first lines of evidence of E6 oncogenic properties come from studies on human tumors cell lineages derived from cervical cancer [45]. Others studies, based on E6 of HPV, pointed out that the oncoprotein is able to induce cytogenetic damage, resulting in genomic instability [29, 30], which is considered a cancer hallmark [46–48]. Although the E6 oncoprotein of both HPV and BPV affects p53, the mechanisms that induce the reduction levels of this tumor suppressor protein are distinct between these PVs types. E6 oncoprotein of HPV binds to the E6AP ubiquitin ligase, resulting in p53 ubiquitination [49]. This process results in p53 proteasomal degradation [50]. However, the BPV-1 E6 oncoprotein of BPV does not induce p53 degradation [51]. Studies pointed out that BPV-1 E6 oncoprotein interacts with CBP/p300, promoting the downregulation of p53 [51]. These different mechanisms of E6 action require studies involving the oncoprotein of BPV.

Mutation is the first step in carcinogenesis process [52]. This study aimed to evaluate the mutagenic potential of E6 recombinant oncoprotein of BPV-1 by the cytokinesis-block micronucleus assay (CBMNA) and comet assay (CA). CBMNA and CA are noninvasive methods, recommended as part of genotoxicity tests battery for drug validation [53]. CBMNA consists in evaluating the frequency of micronucleus in binucleated lymphocytes, obtained through the addiction of cytochalasin B in lymphocyte culture to prevent cytokinesis [54].

Micronuclei (MNs) are acentric fragments expelled from the main nucleus at late stages of anaphase [55]. MNs can be formed through two mechanisms: chromosomal breaks (clastogenesis) or disruption of the mitotic apparatus (aneugenesis) [56]. These fragments remain not integrated in the nucleus of daughter cell, originating the MNs [54, 57]. The MNs represent not only chromosomal losses, but also the result of DNA amplification, commonly observed in oncogenic process [57].

CA is a simple technique, with low cost, which can be employed in any eukaryotic cells [58, 59]. CA has been used to study the clastogenic potential of HPV [60] and BPV [24]. The CMNA combined with CA allows detecting DNA damage as an indication of mutagenesis with high statistical and sensitivity power [53]. This is the first study which evaluated the mutagenic potential of E6 recombinant oncoprotein of BPV-1, suggesting that this oncoprotein participates in both cancer initiation and promotion.

## 2. Material and Methods

### 2.1. Expression and Purification of BPV-1 E6 Oncoprotein

**Figure 1:**
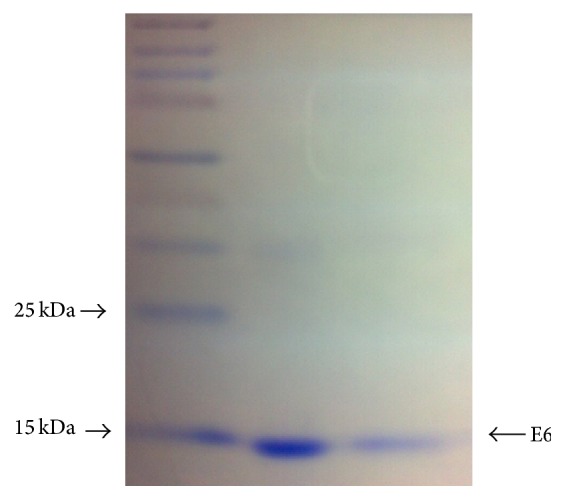
Western blot showing BPV-1 E6 recombinant oncoprotein. Weight ladder employed: Spectra Multicolor Broad Range Protein Ladder (Fermentas, Lithuania).

BPV-1 E6 recombinant oncoprotein was expressed and purified according to Mazzuchelli-de-Souza et al. [44], using* Escherichia coli *BL21. The oncoprotein was subjected to dialysis. This step was necessary to remove urea and imidazole, substances used to promote the oncoprotein refolding. The dialysis avoids false-positive results to mutagenesis tests, which could be induced by urea and imidazole. Dialysis was performed using Slide-A-Lyzer Dialysis Cassette (3 K-12 mL) (Thermo Scientific, Carlsbad, USA). For this step, 10 mL of sample was dialyzed against two liters of dialysis buffer (20 mM Tris-HCl and 500 mM NaCl, pH 8.0) for 8 hours at 4°C under constant agitation.  Figure 1 shows Western blot of BPV-1 E6 recombinant protein used in this study. To confirm the identity of recombinant oncoprotein, the band obtained in the SDS-PAGE gel was submitted to mass spectrometry. Four peptide sequences were identified using this method, being (1) K.DFHVVIR.E, (2) K.DFHVVIR.E, (3) R. HVLFNEPFCK.T, and (4) R.LWQGVPVTGEEAELLHGK.T. These sequences were analyzed in the Swiss-Prot database, where score higher than 43 indicates extensive homology. Thus, mass spectrometry pointed out a score of 94, demonstrating that the recombinant protein shows identity with E6 oncoprotein of BPV-1 (access number VE6_BPV1).

### 2.2. Peripheral Blood Collection

A volume of 5 mL of peripheral blood was collected from five asymptomatic calves (without cutaneous papillomas) (*Bos taurus*, Simmental breed), using the Vacutainer system with EDTA (molecular diagnosis of BPV) and heparin (CBMNA and CA). The material was collected through venipuncture from jugular vein by a veterinarian. The protocols used in this study were approved by the Ethics Committee on Animal Use of Butantan Institute (process 1035/13). The farm of calves' origin is located in São Paulo State (Brazil). This farm was chosen due to the absence of bracken fern* Pteridium aquilinum*, because studies pointed out that bracken farm has mutagenic and carcinogenic compounds, such as quercetin and ptaquilosides [61–63], which could interfere in the studies, leading to false-positive results.

### 2.3. Molecular BPV Identification by PCR

Blood DNA extraction: the extraction of DNA from peripheral blood cells was performed using the GenomicPrep Blood Mini Kit Illustra Spin (GE Healthcare, Buckinghamshire, UK), according to the manufacturer. The quality of the obtained DNA was verified using the polymerase chain reaction (PCR) technique, amplifying a bovine *β*-globin gene fragment [64].

Viral identification: viral identification was performed using specific primers for BPV-1 (forward: 5′-GGAGCGCCTGCTAACTATAGGA-3′; reverse: 5′-ATCTGTTGTTTGGGTGGTGAC-3′), which amplifies the L1 gene, resulting in a 301 bp amplicon, BPV-2 (forward: 5′-GTTATACCACCCAAAGAAGACCCT-3′; reverse: 5′-CTGGTTGCAACAGCTCTCTTTCTC-3′), which amplifies the L2 gene, resulting in a 164 bp amplicon, and BPV-4 (forward: 5′-GCTGACCTTCCAGTCTTAAT-3′; reverse: 5′-CAGTTTCAATCTCCTCTTCA-3′), which amplifies the E7 gene, resulting in a 170 bp amplicon. These primers were chosen because BPV-1, BPV-2, and BPV-4 are the most frequent types in Brazil, being associated with oncogenic process [1, 65]. Besides these specific primers, two degenerate pairs of primers were used:* Delta-Epsilon* (forward: 5′-CCAGAYTAYYTMAAAATGGC-3′; reverse: 5′-ATAAMKGCTAGCTTATATTC-3′) and* Xi *(forward: 5′-TWYAATAGDCCVTTTTGGAT-3′; reverse: 5′-TTMCGCCTACGCTTTGGCGC-3′) [66]. These primers allow detecting BPVs of genera* Delta, Epsilonpapillomavirus *(Delta-Epsilon), and* Xipapillomavirus *(Xi) [66]. Both primers amplify the L1 ORF, resulting in products with 430 bp (Delta-Epsilon) and 600 bp (Xi).

PCR parameters: reactions were performed in a total volume of 50.0 *μ*L, using 200 ng/*μ*L of DNA template, 2.0 *μ*L of forward primer, 2.0 *μ*L of reverse primer, and 36.0 *μ*L of Master Mix (4G, Porto Alegre, Brazil). Reactions were done on thermocycler Veriti 96-Well Thermal Cycler (Applied Biosystems, Carlsbad, USA) and subjected to the cycles shown in Table 2. Cloned genomes of BPV-1, BPV-2, and BPV-4 in pAT153 vector in* Escherichia coli *D5H*α* were used as positive controls. BPV-2 viral genome was employed as positive control for Delta-Epsilon primer and BPV-3 for Xi primer.

The PCR products were analyzed in 2% agarose gel electrophoresis stained with GelRed (Biotium, USA) in Tris-Acetate-EDTA (TAE) buffer, visualized under UV light, using BioDocAnalyze (Biometra, Germany).

### 2.4. Cytokinesis-Block Micronucleus Assay (CBMNA) Using Peripheral Blood Mononuclear Cells (PBMCs)

For each sample, three cultures were established: (1) negative control (not treated with any drug), (2) positive control (treated with 50 *μ*g/mL of cyclophosphamide), and (3) experimental group (treated with 1 *μ*g/mL of E6 recombinant oncoprotein resuspended in PBS). This concentration of E6 recombinant oncoprotein was based on previous study involving BPV early (E) protein as vaccine [67]. The protocol of CBMNA was done according to the technical recommendation proposed by Araldi et al. [53]. In detail, 0.2 mL of peripheral blood was transferred to culture flask containing 5.0 mL RPMI 1640 medium, supplemented with 15% fetal bovine serum, 0.1 mL L-glutamine, and 0.1 mL phytohemagglutinin A. The material was incubated at 37°C for 8 hours. After this, both cyclophosphamide and E6 recombinant oncoprotein were added to the culture. After 44 hours, 0.2 mL of cytochalasin B was added to block the cytokinesis. After 72 hours, the culture was stopped with the addition of 0.5 mL methanol : acetic acid fixative (v/v) (3 : 1) for 5 minutes at room temperature. The material was centrifuged at 500 g and the supernatant was discarded. The pellet was homogenized with 5 mL fixative and centrifuged at 500 g. The pellet was aspirated and transferred to slides, which were stained with a 1 : 3 Giemsa : phosphate buffer solution, pH = 6.8, for 8 minutes.

After staining, coverslips were placed on slides with Entellan (Merck, Germany). The material was analyzed in a blind test under an Axiophot binocular microscope (Carl Zeiss, Germany) to observe the frequency of micronucleated-binucleated lymphocytes in a total of 1000 analyzed cells, according to Araldi et al. [53]. Statistical analysis was performed using the Kruskal-Wallis test followed by a* post hoc* Dunn test, both at a significance level of 5%. Statistical tests were done using the BioEstat software [68].

### 2.5. Comet Assay: Alkaline Method

Slides preparation: slides of 26 × 76 mm were dipped in a solution of normal melting point agarose (NMP) (Invitrogen, Carlsbad, USA) diluted in phosphate-buffered solution (PBS) 1.5% at 60°C, and one side of each slide was wiped clean with a paper towel. The concentration of NMA was based on Araldi et al. [53]. The slides were dried in a horizontal position overnight.

Peripheral blood incubation with drugs: three whole blood aliquots of 200 *μ*L each were transferred to three 1.5 mL polypropylene tubes: (1) negative control, (2) positive control, and (3) experimental group. In each tube, 200 *μ*L of RMPI 1640 medium was added. The negative control did not receive any drug and was incubated only in RPMI 1640 medium. Positive control was incubated with 50 *μ*g/mL of cyclophosphamide diluted in RPMI 1640 medium. Experimental group was treated with 1 *μ*g/mL of BPV-1 E6 recombinant protein. The samples were incubated at 37°C for 2 hours under constant agitation. After this time, the aliquots were centrifuged for 1 minute at 500 g and the supernatant was discarded. Ten microliters of each obtained pellet was added to 75 *μ*L low melting point agarose (LMP). A final volume of 85 *μ*L of this suspension was immediately transferred to NMP precoated slides. The slides were covered with coverslips and maintained at 4°C for 20 minutes. The coverslips were gently removed and the slides were placed in a Coplin jar containing lysis solution (2.5 mM NaCl, 100 mM ethylenediaminetetraacetic acid (EDTA), 10 mM Tris-HCl, 1.1% Triton X-100, and 11.2% dimethyl sulfoxide) at 4°C for 1 hour. Subsequently, all procedures were conducted under dark conditions to prevent the induction of DNA damage.

Electrophoresis: after lysis, slides were washed with PBS and transferred to an electrophoresis tank containing electrophoresis buffer (300 mM NaOH and 1 mM EDTA, pH > 13) at 4°C for 40 minutes to induce unwinding of double-stranded DNA. Next, the electrophoretic run was performed with a current of 25 V (0.86 V/cm), 300 mA, for 20 minutes to promote the migration of free DNA fragments toward the anode. The slides were transferred to a Coplin jar containing neutralizing buffer (400 mM Tris-HCl, pH 7.5) for 5 minutes. The material was fixed in absolute ethanol for 5 minutes.

Comet analysis: slides were stained with 20 *μ*L 4 *μ*g/mL propidium iodide (PI) and analyzed under a fluorescence microscope (Carl Zeiss Axio Scope A1, Germany) equipped with an excitation filter of 510–560 nm and barrier of 590 nm. The material was analyzed in a total magnification of 400x. One hundred nucleoids were analyzed per slide, which were scored on a scale of 0 (without DNA damage) to 2 (maximum DNA damage), according to Araldi et al. [53]. The scores were obtained by summing the product of the observed number of nucleoids per class and its respective class value. Statistical analysis was performed using the Kruskal-Wallis test followed by the* post hoc* Dunn test, both with a significance level of 5%. Both test and graphical analysis were done using the BioEstat software [68].

### 2.6. Cytokinesis-Block Micronucleus Assay (CBMNA) in Epithelial Cells

To verify whether the results observed in PBMCs should also be observed in epithelial cells, CBMNA was performed in CRIB cell (commercial epithelial cell line obtained from bovine kidney). In detail, a total of 1 × 10^5^ cells were transferred to six-well plate, containing a sterile coverslip of 24 × 24 mm with 2 mL of MEM medium, supplemented with 10% fetal bovine serum and 1% ampicillin, and three cultures had been established: negative control (not treated with any drug), positive control (treated with 50 *μ*g/mL of cyclophosphamide), and experimental group (treated with 1 *μ*g/mL of BPV-1 E6 recombinant oncoprotein). Cyclophosphamide and BPV-1 E6 recombinant oncoprotein were added together to the cells. After 1 hour, the three cultures were treated with 6 *μ*g/mL of cytochalasin B (Sigma, Germany). The material was incubated for 48 hours, the time necessary to complete two replication cycles, once the duplication time of these cells is 24 hours, according to our previous study. After this time, the medium was removed and the cells were washed twice with PBS at 37°C. Cells were stained with solution 1 : 4 Giemsa-PBS for 3 minutes and, after, washed twice with PBS. Coverslips containing the biological material were mounted on slides using Entelan (Merck, Germany). Slides were analyzed by Axiophot binocular microscope (Carl Zeiss, Germany) to observe the frequency of micronucleated cells in a total of 1,000 analyzed cells, according to Araldi et al. [53].

## 3. Results

### 3.1. Molecular BPV Identification by PCR

**Figure 2:**
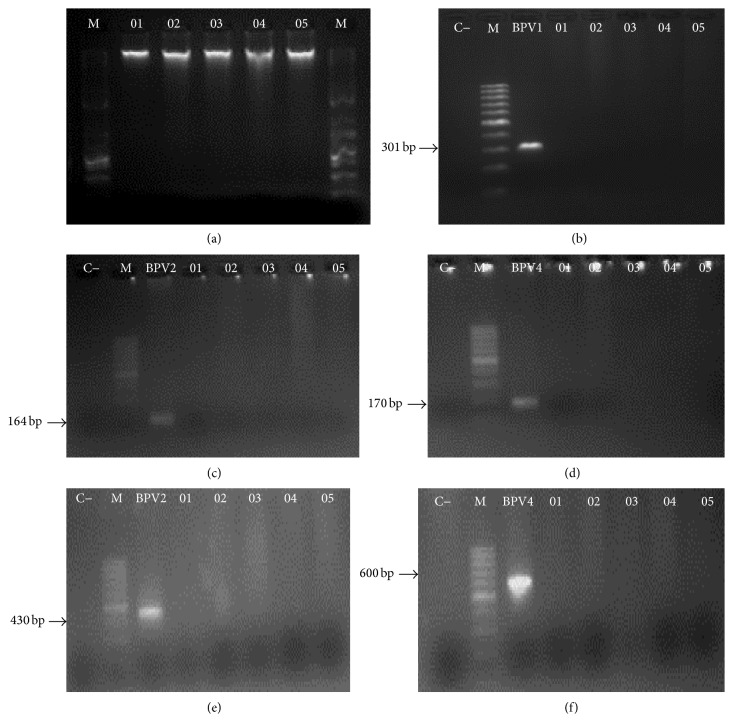
Electrophoresis gel images showing (a) genomic DNA integrity after DNA extraction, using 1 Kb DNA Ladder (Invitrogen, Carlsbad, USA) as marker; absence of amplicon in samples 01 to 05 using specific primers to BPV-1 (b), BPV-2 (c), and BPV-4 (d); absence of amplicon in samples 01 to 05, using Delta-Epsilon (e) and Xi (f) degenerate primers. Images (b)–(f) showed amplification only in positive control, with the 100 bp DNA Ladder (Invitrogen, Carlsbad, USA) being employed as marker.

The peripheral blood samples, collected from five asymptomatic calves, did not reveal the presence of BPV sequences using both specific and degenerate primers (Figure 2).

### 3.2. Cytokinesis-Block Micronucleus Assay (CBMNA) in PBMCs

**Figure 3:**
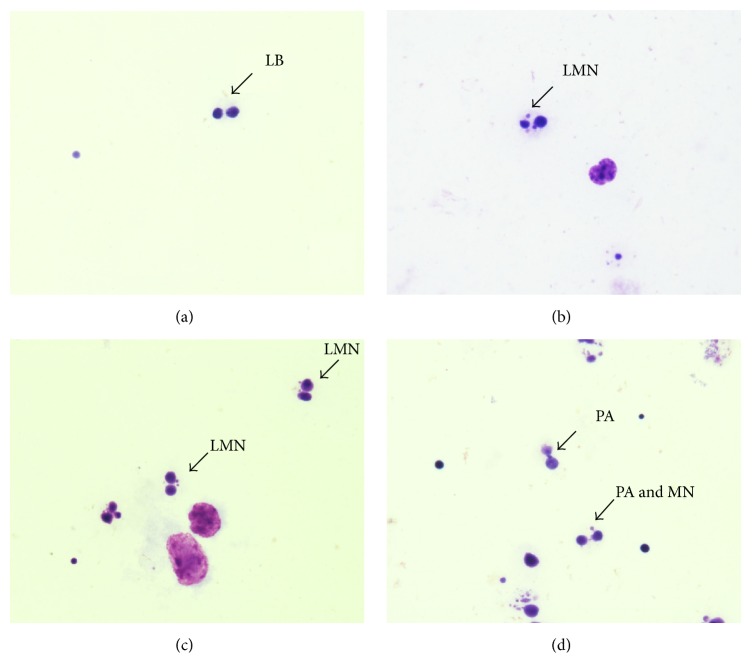
(a) Photomicroscopy of binucleated lymphocyte without micronucleus (LB), observed in negative control. Image of binucleated lymphocyte with micronucleus (LMN), observed in positive control (b) and group treated with E6 recombinant oncoprotein (c). Image of binucleated lymphocyte with anaphase bridge and micronucleus (PA and MN), observed in group treated with E6 (d). Images obtained with total magnification of 1,000x.

Frequency analysis of micronucleus observed in CBMNA: CBMNA showed an elevated number of micronucleated lymphocytes, as well as anaphase bridges, in both positive control and the group treated with 1 *μ*g/mL of E6 recombinant oncoprotein (Table 2, Figure 3).

Based on the micronucleated lymphocytes number observed per group (Table 2), Chi-square (*χ* ^2^) test and Kruskal-Wallis test were done, both with 5% significance level. Chi-square test pointed out statistical differences between positive control and group treated with E6 (Table 3). These results point out that the cyclophosphamide and E6 recombinant oncoprotein are able to induce aneugenesis and/or clastogenesis. However, the Chi-square test did not show statistic difference in negative control (Table 3).

Kruskal-Wallis test revealed statistical significant differences among the groups (*H* = 9.7297 and *p* = 0.0087). Based on this result, Dunn* post hoc *test was performed. The test pointed out significant difference between negative and positive control, as well as negative control and experimental group (E6) (Table 4). However, the test did not show statistical difference between positive control and experimental group (Table 4).

**Figure 4:**
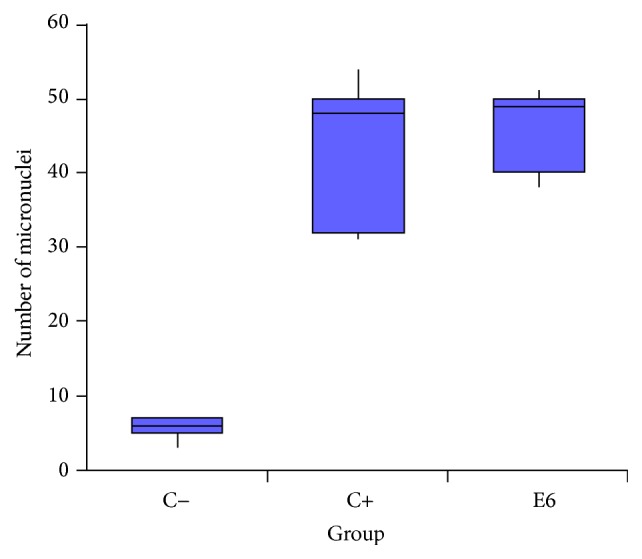
Comparative boxplot, based on maximum, minimum, and median values of micronucleus observed per group.

Based on the maximum, minimum, and median values of micronucleus observed in the three groups, a boxplot was done (Figure 4). The graph indicates statistically equal medians between positive control and experimental group (E6). Although the medians between these groups had not shown statistical differences, the graph indicates that the experimental group showed maximum values of binucleated lymphocytes with micronucleus higher than positive control.

Frequency analysis of anaphase bridge observed in CBMNA: based on the number of anaphase bridges (AB) observed per group (Table 3), Kruskal-Wallis test was performed, which indicates significant differences among the groups (*H* = 8.3444, *p* = 0.0154). Student-Newman-Keuls* post hoc *test revealed differences between negative and positive control, as well as negative control and experimental group (Table 5). The test did not show differences between positive control and experimental group.

Analysis of cytokinesis-block proliferation index (CBPI) and cytotoxicity: Kruskal-Wallis test, based on the CBPI, indicates statistical differences among the groups (*H* = 9.3968 and *p* = 0.0091). Dunn* post hoc *test revealed significant differences between negative and positive control, as well as negative control and experimental group (Table 6). However, the test did not show differences between positive control and experimental group. These data indicate that E6 recombinant oncoprotein has CBPI similar to the cyclophosphamide.

**Figure 5:**
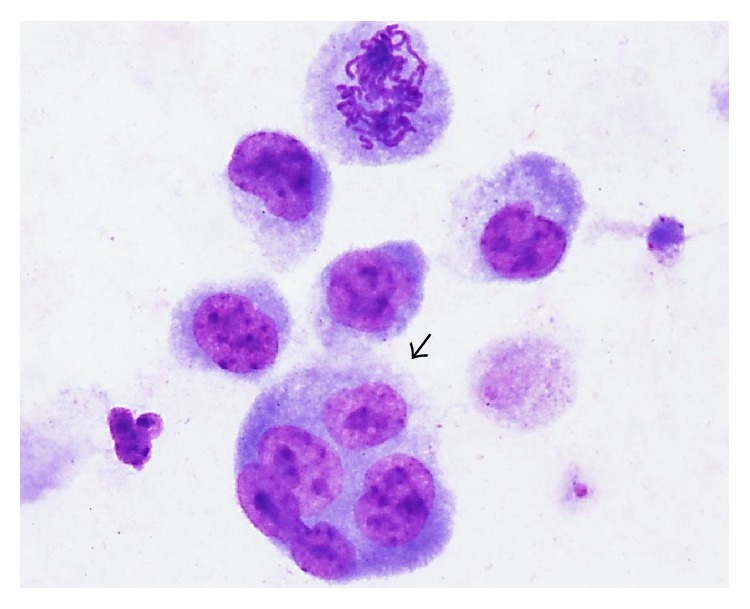
Evidence of neosis. Photomicroscopy of lymphocyte showing endoreduplication (black arrow), suggesting neosis. Cells analyzed in total magnification of 1,000x.

Besides presenting high CBPI, the E6 recombinant oncoprotein also showed to induce endoreduplication, evidenced by the presence of intracellular cytokinesis, suggesting neosis (Figure 5), according to the criteria proposed by Das et al. [69]. The CBMNA results suggest that E6 recombinant oncoprotein induces mitotic stress, resulting in clastogenesis and neosis.

### 3.3. Comet Assay: Alkaline Method

**Figure 6:**
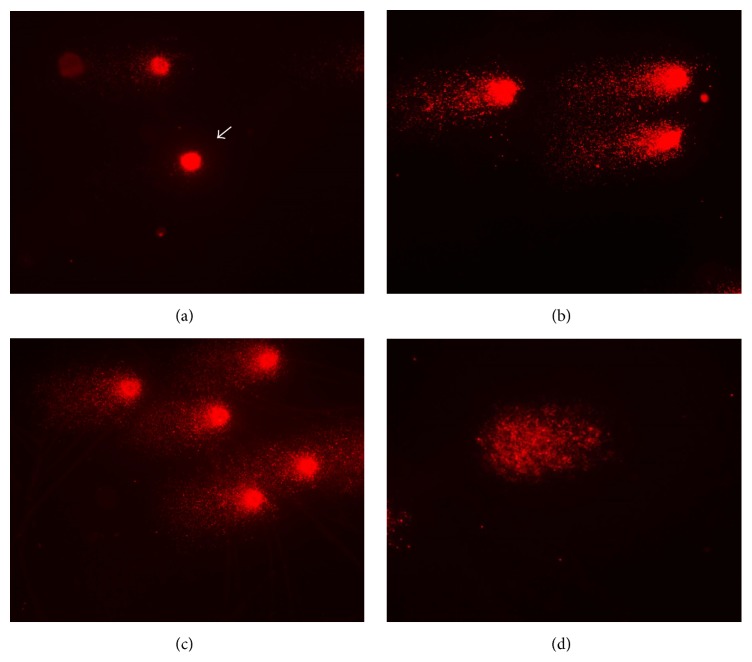
Images of comets' nucleoids. (a) Image of class 0 (without DNA damage) observed in negative control group. Images of class 2 (maximum DNA damage) observed in positive control (b) and experimental (c) groups. (d) Image of hedgehog comet, characterized by the head absence, observed in experimental group. Images captured in total magnification of 400x.

**Figure 7:**
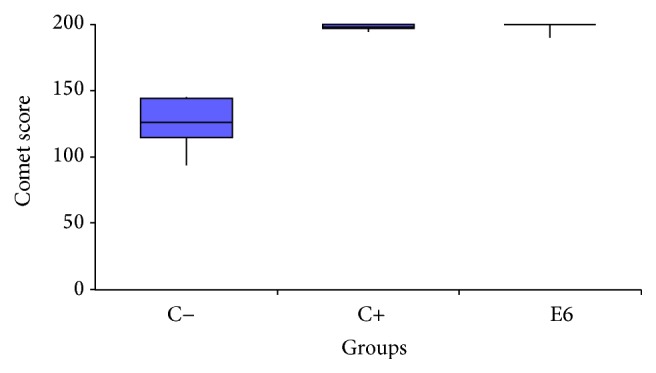
Boxplot of comet score.

Comet assay results are shown in Table 7 and Figure 6. According to these data, the Kruskal-Wallis test was performed, which pointed out statistical differences among the groups (*H* = 10.2613 and *p* = 0.0059). Based on these results, the Dunn* post hoc *test was performed, which pointed out significant differences between negative and positive control, as well as negative control and experimental group (Table 8). However, the test did not show differences between positive control and experimental group (Table 8). These results were also verified by Figure 7. Comet assay reinforces the CBMNA results, indicating the mutagenic potential of E6 recombinant oncoprotein.

### 3.4. Cytokinesis-Block Micronucleus Assay (CBMNA) in PBMCs

**Figure 8:**
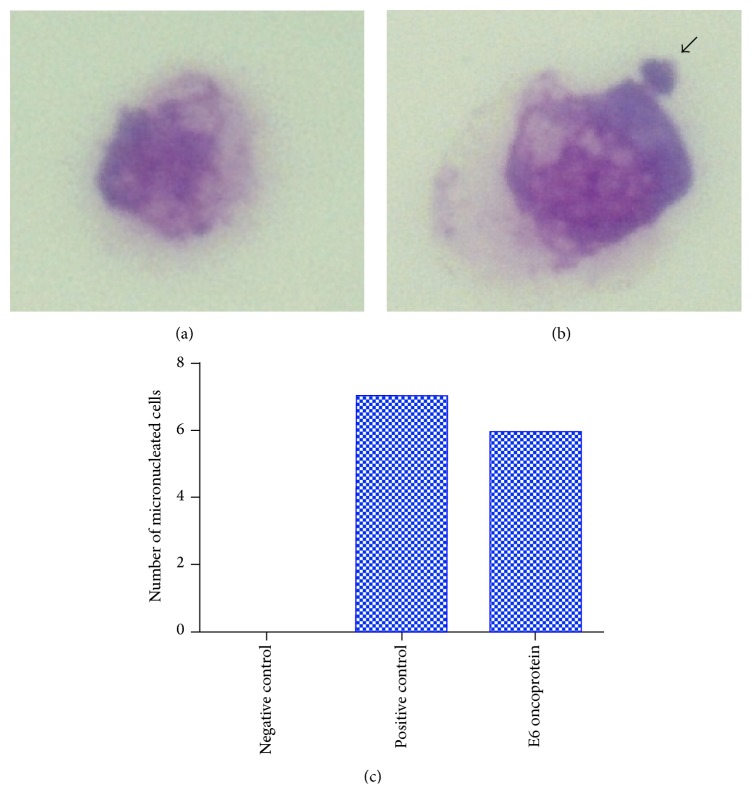
Photomicroscope showing CRIB cell not treated with any drug (negative control), indicating absence of micronucleus (a), and treated with BPV-1 E6 recombinant oncoprotein, showing the presence of MN, indicated by arrow (b). (c) Number of micronucleated cells observed in each group.

Results of this test confirmed the previous results, indicating that the BPV-1 E6 recombinant oncoprotein has a genotoxicity potential, being able to induce DNA breaks in epithelial cells, verified by the presence of MNs (Figure 8).

## 4. Discussion

Transforming potential of E6 oncoprotein has been discussed since the 1980s, based on studies of cottontail rabbit papillomaviruses (CRPV) [70, 71]. Although there are lines of evidence that the E6 oncoprotein can induce transformation [72], the mechanisms of initiation and promotion of cancer associated with the BPV E6 oncoprotein are unknown.

Molecular diagnosis of five peripheral blood samples from five calves showed the absence of amplicons for the five different primers used (Figure 2). However, both specific (BPV-1, BPV-2, and BPV-4) and degenerate (Delta-Epsilon and Xi) primers sets amplified the controls genomes of BPV-1, BPV-2, and BPV-4. None of the negative controls revealed the amplicon presence. These results indicate that the five samples were uninfected by BPV. Moreover, the use of specific and degenerate primers increases the capacity of BPV identification. This occurs because specific primers are more sensitive than degenerates [66]. This sensitivity is due to the absence of degeneration in the 5′ region, which reduces the primer ability to recognize and to link with DNA target sequence [73]. The absence of BPV infection in the peripheral blood samples is required for the CBMNA and CA, allowing investigating the aneugenic and/or clastogenic action of BPV-1 E6 recombinant oncoprotein. This is because the presence of BPV sequences is associated with the presence of oncoproteins transcripts [74]. Thus, studies have shown that the presence of BPV DNA sequences in peripheral blood is associated with cytogenetic damage, including clastogenesis [1, 14, 75]. Complementary studies show the association of the BPV presence with cytogenetic damage in both benign lesions (papillomas) [74] and carcinomas [15].

Chi-square test, based on the number of micronucleated cells, pointed out significant statistical values in both positive control and group treated with BPV-1 E6 recombinant oncoprotein (Table 3). Negative control showed fewer number of micronucleated cells. This result was expected, because the biological material was transported and processed after three hours of its collection. This procedure reduced the influence of exogenous environmental factors, which could induce DNA damage. Furthermore, the absence of BPV infection in the peripheral blood reduces the influence of endogenous environmental factors that could interfere in the analysis. Positive control showed a high number of micronucleated lymphocytes. This result was also expected, once this group was treated with 50 *μ*g/mL of cyclophosphamide, a chemotherapeutic drug with cytotoxic and teratogenic effect [76, 77]. The cells treated with 1 *μ*g/mL of BPV-1 E6 recombinant oncoprotein also showed significant statistical values. Kruskal-Wallis test, followed by the Dunn* post hoc *test, was performed to compare the frequencies of micronucleus among three groups. This test indicated statistical difference between negative control and the cells treated with BPV-1 E6 recombinant oncoprotein. However, the test did not reveal differences between the cells treated with cyclophosphamide and BPV-1 E6 recombinant oncoprotein (Table 4, Figure 4). Similar results were also observed in epithelial cells (Figure 8). These results suggest that the BPV-1 E6 oncoprotein has an aneugenic and/or clastogenic potential similar to or higher than the observed with the cyclophosphamide. This is because the cells treated with the oncoprotein showed a frequency of micronucleus formation (MN_*r*0_) 14,003% higher than those observed in positive control.

The elevated mutagenic potential of the BPV-1 E6 oncoprotein can justify the cytogenetic damage already described in the literature [1, 14, 15, 24]. Similar results were observed in HPV-infected cells [12, 78–81]. The micronucleus inducement was already described in cytological samples, collected during Papanicolau's test, from healthy cervix infected by HPV [12]. For this reason, the micronucleus assay has been proposed as a complementary method for Papanicolau's test, being a suggestive biomarker of lesion degree [78]. However, the association between the BPV and the micronucleus inducement was not yet reported.

The high frequency of micronucleated lymphocytes in samples infected by HPV has been attributed to the synergic action of E5, E6, and E7 oncoproteins [29, 30, 78, 79]. However, this is the first study that pointed out the mutagenic action of BPV-1 E6 protein* per se.*

Student-Newman-Keuls* post hoc *test, based on the frequency of anaphase bridges, showed significant statistical difference between the negative control and the group treated with the BPV-1 E6 recombinant oncoprotein (Table 5). However, the test did not reveal differences between the cells treated with cyclophosphamide and BPV-1 E6 recombinant oncoprotein (Table 5). These data reinforce those observed by the Dunn* post hoc *test, based on the frequency of micronucleus, indicating the clastogenic action of the E6 oncoprotein. Moreover, the presence of anaphase bridge was already described in cells transfected with recombinant adenovirus containing the E6 and E7 ORFs of HPV [29, 30]. However, there are no studies describing the action of BPV E6 oncoprotein in the induction of anaphase bridges or studies that show this same action of E6 oncoprotein of HPV* per se*. Anaphase bridges are indicators of genomic instability [82, 83], which is considered the first step in carcinogenesis [46, 47]. Moreover, the presence of these bridges is an important hallmark of DNA double strand breaks (DSBs) [84].

Statistical analysis, based on the cytokinesis-block proliferation index (CBPI), showed that BPV-1 E6 oncoprotein has cytotoxic levels similar to cyclophosphamide (Table 6). This result suggests that the BPV E6 oncoprotein can deregulate the cell cycle, contributing to the cell proliferation and immortalization. To date, this mitogenic action is attributed to the E7 oncoprotein [85–87]. E7 oncoprotein induces the phosphorylation of pRb, resulting in the E2F transcription factor release [85]. E2F, when translocated to the nucleus, acts as activator and binds to the kinase-dependent cyclins promoters [85]. However, the results showed polynucleated cells in the group treated with BPV E6 oncoprotein (Figure 5), suggesting that the BPV E6 oncoprotein confers mitogenic stimulation, resulting in mitotic stress. Moreover, studies pointed out that the BPV E6 oncoprotein interacts with the CBP/p300 deacetylase, promoting p53 downregulation [88]. This downregulation increases the expression levels of FoxM1 transcription factor (*Foxhead box M1*) [89], promoting B1 cyclin, D1 cyclin, and cdc25 upregulation [89]. The upregulation of these genes is associated with increased levels of cell proliferation, which is necessary in order to make DNA polymerases available to virus replication. However, if this mechanism guarantees the BPV replication, it can contribute to cell immortalization and cancer progression.

The FoxM1 factor also participates in the Wnt/*β*-catenin signaling pathway and binds directly to the *β*-catenin [89]. This interaction promotes the nuclear *β*-catenin translocation [90]. *β*-catenin translocation to nucleus induces the cyclin expression [64]. So, these data suggest that the BPV E6 oncoprotein not only promotes mitogenic stress, but also contributes to the epithelial-mesenchymal transition (EMT). Studies based on the HPV E6 oncoprotein have indicated that this oncoprotein contributes significantly to the EMT [91]. This occurs not only because the E6 oncoprotein induces the translocation of *β*-catenin, but also due to the proteasomal degradation of regulatory proteins of apical-basal polarity [92–94]. Studies also showed that the E6 oncoprotein is able to bind to* Crumbs* (*Dlg *and* Patj*) proteins, resulting in loss of cell polarity [93, 94]. Based on these results, the E6 oncoprotein emerges as a possible therapeutic target with biotechnological value in cancer treatment.

Mitogenic action of E6 oncoprotein can be more expressed in cells infected by papillomaviruses, once these cells also expressed E5 and E7 oncoproteins [74]. The E7 oncoprotein is able to form a complex E7-p600, which promotes E6 upregulation [85]. Furthermore, the E6 oncoprotein can bind to the E6AP ubiquitin ligase, forming the E6-E6AP complex, increasing the* hTERT *levels of expression, contributing to cell immortalization [95]. Moreover, E6-E6AP induces NFX1 expression, promoting MHC-II downregulation [96]. This mechanism reduces the antigenic presentation mediated by CD4+ T lymphocytes, contributing to immune evasion. This action guarantees the PV infection for long periods with or without clinical symptoms [24]. Although the BPV infection can be asymptomatic, the BPV presence in peripheral blood is associated with DNA damage [1, 14, 24].

CBMNA analysis pointed out the presence of cells with intracellular cytokinesis (Figure 5). This result suggests the neotic action of BPV E6 oncoprotein. Neosis is characterized by the presence of (1) DNA damage, (2) loss of checkpoint control, (3) repair system failures, and (4) endoreduplication [97]. In this scenario, the E6 oncoprotein attempts all these neosis criteria. Moreover, the oncoprotein not only increases the cytogenetic damage, leading to increasing of micronucleus frequency, but also induces clastogenesis, as shown by the comet assay and anaphase bridges. So, statistical analysis performed based on the comet scores reinforced the CBMNA results. Comet assay allows detecting both simple and double DNA breaks, being more sensitive than the CBMNA. Clastogenesis is considered the most serious type of DNA damage [98]. DNA breaks can be repaired by homologous or nonhomologous recombination. However, studies point out that the E6 oncoprotein can link to the Holliday junction during the homologous repair, avoiding the junction resolution [99]. Moreover, E6 oncoprotein promotes the* TP53* gene deacetylation, resulting in p53 downregulation [51]. This epigenetic effect results in cell cycle deregulation, affecting the* checkpoints*.

Studies have shown that the HPV-induced DNA breaks are required for the virus integration into the host genome [100]. Although there are no studies showing the BPV integration to date, the results observed in this work, in addition to those already published [24], indicate the necessity of more studies to evaluate the virus-host interaction.

Studies also showed that the E6 oncoprotein induces clathrin-transporter adapter protein (AP-1), increasing the level of this protein in the plasma membrane [76, 77]. The increasing quantity of AP-1 in membrane can facilitate the BPV virions infection. This occurs because the infection process is clathrin dependent. In this scenario, the E6 oncoprotein can contribute to the virus infection [101].

In summary, the CBMNA and CA results showed that the BPV E6 oncoprotein participates not only in cancer promotion, but also in initiation, inducing DNA breaks. These DNA breaks represent mutagenic effects, considered the first step in the oncogenesis process, which are associated with genomic instability [47, 48, 102, 103]. Thus, the E6 recombinant oncoprotein has been suggested as a possible vaccine candidate [104–112] due to its immunogenicity [44]. However, the mutagenic tests, such as CBMNA and CA, are required in drug validation process [113]. In this scenario, this study pointed out that the BPV-1 E6 recombinant oncoprotein, in tested concentration, showed mutagenic potential. This is the first study that reports the mutagenic potential of a possible therapeutic vaccine candidate. On the one hand, these results allowed better understanding the mechanism of cancer promotion associated with the BPV E6 oncoprotein, as well as revealing that this oncoprotein can induce carcinogenesis* per se*; on the other hand, this data pointed out that maybe BPV E6 recombinant oncoprotein requires protein modifications to be used as vaccine.

## Figures and Tables

**Table 1:** Studies already published about E6 oncoprotein.

Interaction with host cell	Virus type	Method	References
Facilitating virus entry	BPV	E6 facilitates the BPV entry by clathrin interaction	[35]

Antiapoptotic effect	HPV	E6 promotes *Bax* degradation, resulting in an antiapoptotic effect	[114, 115]

Binding to E6AP ubiquitin ligase	HPV	E6 expressed in *E. coli *and insects binds to E6AP ubiquitin ligase	[50, 95, 116, 117]

Binding to DNA	HPV	E6 expressed in *E. coli* binds with DNA	[118–120]

Cell immortalization	HPV	E6 promotes p53 degradation and cell immortalization	[121–123]

Epigenetic downregulation of p53	HPV	E6 expressed in baculovirus induces hypoacetylation of p53	[124, 125]
	HPV	E6 expressed in *E. coli * interacts with p300/CBP, reducing p53 levels	[126]
	BPV	E6 interacts with p300/CBP, reducing p53 levels	[51]

Disruption of mitotic apparatus	HPV	E6 transfected using LXSN vectors	[29, 30, 127]

Malignant transformation in cell culture	HPV	Complete genome of HPV-16 transfected in NIH 3T3 cells	[45, 88, 128–130]

Interaction with paxillin	BPV	E6 of BPV-1 interacts with paxillin, reducing the focal adhesion	[34, 69, 72, 131, 132]

Repressor activity of telomerases	HPV	E6 induces the hTERT expression, reducing the telomerase activity	[96, 133, 134]

Immune depletion	HPV	E6 expressed in yeast reduces the levels of interferon regulatory factor-3	[135]

**Table 2:** Frequency of micronucleated lymphocytes. Number of micronuclei observed perslide (MN) and number of mononucleated (*N*_1_), binucleated (*N*_2_), and polynucleated (*N*_*p*_) lymphocytes and anaphase bridges (AB) observed pergroup. Based on these values, the micronucleated formation frequency (MN_*r*0_) and the cytokinesis-block proliferation index (CBPI) and the media ($\overset{-}{x}$) are shown.

Sample	MN	*N*_1_	*N*_2_	*N*_*p*_	AB	MN_*r*0_	CBPI
Negative control							
01	7	954	22	24	3	0.3181	1.070
02	5	963	19	18	3	0.2631	1.055
03	6	949	22	29	0	0.2727	1.051
04	3	953	17	30	1	0.1764	1.077
05	7	960	22	18	5	0.3181	1.058
Total	**28**	**4,779**	**102**	**119**	**12**	$\overset{-}{x}=0.2696$	$\overset{-}{x}=1.062$

Positive control (cyclophosphamide)							
01	32	906	51	43	6	0.6274	1.137
02	31	873	62	65	8	0.5000	1.192
03	48	890	70	40	7	0.6857	1.150
04	54	903	62	35	14	0.8709	1.132
05	50	875	52	73	4	0.9615	1.198
Total	**215**	**4,447**	**297**	**256**	**39**	$\overset{-}{x}=0.7291$	$\overset{-}{x}=1.161$

BPV-1 E6 recombinant oncoprotein							
01	51	892	60	48	4	0.8500	1.156
02	50	894	74	32	9	0.6756	1.138
03	49	900	53	47	10	0.9245	1.147
04	40	901	41	58	10	0.9756	1.157
05	38	905	43	52	10	0.7307	1.147
Total	**228**	**4,492**	**271**	**237**	**43**	$\overset{-}{x}=0.8312$	$\overset{-}{x}=1.149$

**Table 3:** Chi-square (*χ* ^2^) test results, showing expected value (*E*), freedom degree (FD), and probability (*p*).

Group	*E*	*χ* ^2^	FD	*p*
Negative control	5.60	2.000	4	0.7358
Positive control	43.0	10.698	4	0.0302
Experimental (E6)	45.6	12.202	4	0.0159

**Table 4:** Dunn *post hoc *test results.

Groups	Rank differences	Calculated *Z*	Critical *Z*	*p*
C− and C+	7.1	2.5102	2.394	<0.05
C– and E6	7.9	2.7931	2.394	<0.05
C+ and E6	0.8	0.2828	2.394	n.s.

n.s.: nonsignificant value.

**Table 5:** Student-Newman-Keuls *post hoc* test results.

Groups	Rank differences	Calculated *Z*	Critical *Z*	*p*
C− and C+	6.1	2.1567	2.394	0.0310
C− and E6	7.7	2.7224	2.394	0.0065
C+ and E6	1.6	0.5657	2.394	0.5716

**Table 6:** Dunn *post hoc *test results based on the cytokinesis-block proliferation index.

Group	Rank differences	Calculated *Z*	Critical *Z*	*p*
C− and C+	7.6	2.6870	2.394	<0.05
C− and E6	7.4	2.6163	2.394	<0.05
C+ and E6	0.2	0.0707	2.394	n.s.

n.s.: nonsignificant differences.

**Table 7:** Comet assay results, showing the number of nucleoids observed per class, the number of hedgehog comets, and comet score.

Sample	Class 0	Class 1	Class 2	Hedgehog	Score
Negative control					
01	9	37	54	4	145
02	19	36	45	3	126
03	10	86	4	4	94
04	2	52	46	2	144
05	3	79	18	0	115

Positive control (cyclophosphamide)					
01	0	2	98	5	198
02	0	3	97	3	197
03	3	0	97	8	194
04	0	0	100	8	200
05	0	0	100	6	200

BPV-1 E6 recombinant oncoprotein					
01	2	6	92	8	190
02	0	0	100	0	200
03	0	0	100	6	200
04	0	0	100	7	200
05	0	0	100	10	200

**Table 8:** Dunn *post hoc *test results of comet assay.

Groups	Rank differences	Calculated *Z*	Critical *Z*	*p*
C− and C+	6.8	2.4042	2.394	<0.5
C− and E6	8.2	2.8991	2.394	<0.5
C+ and E6	1.4	0.4950	2.394	n.s.

n.s.: nonsignificant differences.
